# Mr. Cuming, in Reply to Academicus

**Published:** 1804-06-01

**Authors:** Ralph Cuming

**Affiliations:** Romsey


### *To the Editors of the Medical and Physical Journal.*

GENTLEMEN,

IN compliance with the desire of Academicus, I have endeavoured to elucidate the case of the little boy, noticed in p. 461 of your last Number. The remote cause of his illness may, I think, be attributed solely to the effects of cold; which certainly would not have affected him half so severely, if he had breakfasted prior to his exposure. Though there is nothing in the short history of the case, which could lead one to suppose that the child suffered in a peculiar manner from the situations in which he was placed, either before or after breakfast; yet he might, unobserved, have been exposed to a current of air. Though delicacy almost forbids me to animadvert on on the means adopted for the recovery of the child, yet a sense of duty imperiously stifles inferior considerations, and declares the propriety of dissenting in opinion, and of pointing out such remedies as might have been conducive to what the parents would have considered a happy event.

Then, advancing on the position that cold was the remote or occasional cause of the child's illness, would not an emetic have had a good effect in overcoming that stricture on the surface, which was certainly the proximate cause of the disease. The perspiration was checked, and those fluids which should have escaped by the skin, were determined to the bowels, producing that congestion which was certainly the cause of bilious dejections. When the apothecary was sent for, I suppose, from his sending a saline mixture, that the child had some sickness at the stomach; the calomel purge was well calculated to rid the bowels from bilious matter, and the blister might have some effect in relieving sickness.

When the physician was called in, he continued the calomel, and ordered blisters to the inside of each leg; his ideas of treatment seemed to coincide perfectly with those of the apothecary. In such cases as these, I have found the happiest effects from emetics in the first instance, followed up by sudorifics; and by way of an auxiliary, I have often had recourse to the warm bath, keeping the child in it until it became faint, after which it has been wiped dry, and wrapped up in flannel, which will in general restore the perspiration, the first grand step towards recovery. That connection which subsists between the bowels and skin is very striking; when the pores are obstructed, diarrhœa or fever, for the most part, supervenes, and is frequently attended with sickness or vomiting; and Sydenham tells us, that these complaints are not to be overcome without the aid of external means, applied for the purpose of producing perspiration, or a determination to the surface of the body. I am of opinion, if the warm bath was resorted to oftener than it is, the art of healing would be greatly benefited. In bilious cases, when the remote causes may be attributed to the effects of cold, and when there is a congestion of blood in the vena portarum and hepatic vessels, does not the warm bath appear to be a remedy likely to be productive of the happiest consequences? In cholera morbus, a disease unparalleled for its violence, and which brings many to an untimely grave, this remedy might be employed with great advan-

L 1 4 tage.

tage. In all fevers, during the state of apyrexia, we find the stomach is relieved from nausea; and when the perspiration is restored, the different parts of the body perform their functions with ease.

There is another remedy for restoring perspiration, which I have employed myself in several instances, and particularly in typhus, or ship fever. I once had a patient, whose fever had continued many days without any intermission, during a voyage to America. I had recourse to such medicines as are generally used on these occasions, without experiencing the least benefit from them; his skin was parched, tongue foul, black, and dry, with every unfavourable symptom. I ordered him to be taken out of his hammock, had his shirt taken off, and several buckets of cold water poured over his head, gradatim, for it was necessary to be cautious in this case, as he was extremely debilitated, and had been delirious for some time; he was wiped dry, wrapped up in blankets, and put to bed again. The cold applied to his head, soon restored the lost energy of the brain, and reason again assumed her throne. The effects of the cold on his body, produced such a degree of reaction, that the stricture of the extreme vessels was easily overcome; he burst out into an abundant perspiration; and from this moment he began to recover, and his cure went on rapidly.

It is well known how difficult it is to prevail upon young children to take medicine; and when administered, its inefficacy, unaided by other means, is too frequently experienced: and where it is obvious, from a review of the occasional causes of a disease, that cuticular secretion forms the most prominent and leading indication of cure, what is more likely to effect this, than the tepid bath? In the hot fit of an intermittent, when the patient is scorched as it were with fever, how sensibly is he relieved when the pores of the skin are forced open, and perspiration begins to flow. But here I must remark, in allusion to that mode of treatment which I have recommended, that many of our first-rate physicians have had candour enough to acknowledge, after the death of their patient, that a different treatment might have crowned their endeavours with success; yet such declarations are not clothed with the garb of conviction. Medical men are certainly instrumental towards the recovery of people from sickness; but when the decree of death is passed, the most splendid abilities lose their lustre amidst the dark gloom of expiring nature!

nature! The fatal symptoms of coma and convulsions, which closed the scene of this child's illness, might have been occasioned by hydrocephalus.

I am, &c.

*Romsey, May* 11, 1804. RALPH CUMING.

